# Premature infants receiving delayed cord clamping with and without cord milking: a randomized control trial

**DOI:** 10.1186/s12887-023-03933-2

**Published:** 2023-03-18

**Authors:** Sariya Prachukthum, Chamnan Tanprasertkul, Charintip Somprasit

**Affiliations:** 1 Department of Pediatrics, Faculty of Medicine, Thammasat University, Pathumthani, 12120 Thailand; 2 Department of Obstetrics and Gynaecology, Faculty of Medicine, Thammasat University, Pathumthani, Thailand

**Keywords:** Delayed cord clamping, Placental transfusion, Preterm infants, Umbilical cord milking

## Abstract

**Background:**

Preterm infants often have long hospital stays and frequent blood tests; they often develop anemia requiring multiple blood transfusions. Placental transfusion via delayed cord clamping (DCC) or umbilical cord milking (UCM) helps increase blood volume. We hypothesized umbilical cord milking (UCM), together with DCC, would be superior in reducing blood transfusions.

**Objectives:**

To compare the effects of DCC and DCC combined with UCM on hematologic outcomes among preterm infants.

**Methods:**

One hundred twenty singleton preterm infants born at 280/7- 336/7 weeks of gestation at Thammasat University Hospital were enrolled in an open-label, randomized, controlled trial. They were placed into three groups (1:1:1) by a block-of-three randomization: DCC for 45 s, DCC with UCM performed before clamping (DCM-B), and DCC with UCM performed after clamping (DCM-A). The primary outcomes were hematocrit levels and number of infants receiving blood transfusions during the first 28 days of life. Intraventricular hemorrhage (IVH) and necrotizing enterocolitis (NEC) were secondary outcomes. Analyses were performed with an intent-to-treat approach.

**Results:**

One hundred twenty preterm infants were randomized. There was no statistically significant difference in neonatal outcomes; hematocrit on admission 54.0 ± 5.5, 53.3 ± 6.0, and 54.3 ± 5.8 (*p* = 0.88), receiving blood transfusions 25%, 20%, and 12.5% (*p* = 0.24), incidence of NEC 7.5, 0 and 10% (*p* = 0.78) in the DCC, DCM-B and DCM-A groups, respectively. There were no preterm infants with severe IVH, polycythemia, maternal or neonatal death.

**Conclusion:**

The placental transfusion techniques utilized, DCC and DCC combined with UCM, provided the same benefits for preterm infants born at GA 28 and 33 weeks in terms of reducing the need for RBC transfusions, severities of IVH and incidence of NEC without increasing comorbidity.

**Trial registration:**

TCTR20190131002. Registered 31 January 2019—Retrospectively registered.

## Background

Currently, performing delayed cord clamping (DCC) is the standard care practice recommended for preterm infants by many professional organizations [1, 2]. Preterm infants receiving a placental transfusion have increased circulating blood volume, thereby improving blood pressure, less necessary blood transfusions, and lower incidence of severe intraventricular hemorrhage (IVH) and necrotizing enterocolitis (NEC) [3–7]. The American College of Obstetricians and Gynecologists recommend DCC 30–60 s for preterm deliveries [5]. For the infants’ position, infants who were placed on the maternal abdomen or chest received the same amount of blood from DCC as holding the infants at the level of the vagina [8, 9].

In preterm infants, umbilical cord milking (UCM) has the same benefits as DCC in terms of red blood cell (RBC) transfusion requirements [10, 11]. A meta-analysis suggests that DCC or UCM both have advantages over immediate cord clamping regarding decreased blood transfusion incidence, decreased overall mortality, and lower risk of intraventricular hemorrhage [12]. Currently, UCM should not be performed in extremely preterm infants due to UCM significantly increasing the incidence of severe IVH more than DCC [13–15].

In a study of preterm infants born by cesarean section, the DCC group had a blood volume greater than the early cord clamping group [16]. However, it was not significantly different, and this may be because the umbilical arteries still have blood flow from the infant to the placenta; umbilical vein blood flow is bidirectional. When or if the infant cries, blood from the infant flows back to the placenta [16]. Hence, we hypothesized that preterm infants who underwent DCC combined with UCM, before or after umbilical cord clamping, may receive more blood volume than those who had DCC alone. The aim of our study was to assess the effect of DCC and DCC combined with UCM on the number of infants needing RBC transfusions during the first 28 days of life, and the prevalence of IVH and NEC.

## Methods

Preterm infants born at GA 28^0/7^- 33 ^6/7^ weeks at Thammasat University Hospital, Pathumthani, Thailand, between July 1^st^, 2016, and December 31^st^, 2018 were enrolled in a randomized controlled trial by the delivery nursing team. We excluded infants who were multiples, diagnosed with severe disabilities, having chromosomal abnormalities, hydrops fetalis, intrauterine growth retardation, or from mothers with placenta previa with hemorrhage, abruption placenta, prolapsed cord, or having fetal distress before birth, as well as those mothers who were giving birth before or on arrival, or unable to give consent. Of note, after enrollment, no infant had fetal distress. This study was approved by the Human Research Ethics Committee No.1, Faculty of Medicine, Thammasat University. The informed consent was obtained before delivery by our research staff.

### Randomization

Participants were divided into three groups (1:1:1) by a block-of-three randomization using sealed envelopes, performed by the research team. Envelopes were opened by the delivery nursing team, when mothers commenced preterm labor and had at least four regular uterine contractions in 20 min with cervix dilatation > 4 cm or when mothers had preterm birth scheduled due to severe preeclampsia.

### Placental transfusion techniques

DCC means delaying cutting the umbilical cord after birth for 45 s. DCM-B is DCC for 45 s with an obstetrician then performing UCM on about 25 cm length of the cord three times, then cutting the umbilical cord. DCM-A is DCC for 45 s with the umbilical cord cut to around 25 cm length; after this, the pediatrician milks the umbilical cord toward the infant three times, and the cord is cut to standard. Digital clocks were used to time the procedure.

During these interventions, infants born by cesarean section were placed on the maternal abdomen, and for those born by vaginal delivery, the obstetrician held the infant at the maternal vaginal level.

After cutting the cord, the preterm infants were wrapped in a plastic bag and placed on a warm mattress under the radiant warmer. Neonatal resuscitation was decided by the attending physician at birth.

All infants received our standard care. Our feeding protocol started when the infant had been stable for at least 24 h, then trophic feeds of 10–15 ml/kg/day were initiated for two days, and advanced in increments of 15–20 ml/kg/day. After the infant could tolerate 120 ml/kg/day of milk for two days at 12–14 days of age, a starter iron supplement of 4 mg/kg/day was given. In addition, an ultrasound brain screening was performed between 14 and 28 days of life by a pediatric radiologist who was blinded to the intervention. Jaundice was assessed using serum microbilirubin, and verification of hematocrit levels within one hour of birth was checked at 1^st^ and 4^th^ week of age. If an infant was discharged home before 28 days of life, there was an appointment to follow up and check hematocrit levels at the 4^th^ week of age.

### Outcomes

All outcomes were analyzed by blinded team researchers who did not know the code of the intervention. The primary outcome was preterm infants received RBC transfusions within 28 days of age. RBC transfusion was given 10–15 ml/kg for preterm infants who had one of these 3 conditions: I. infants requiring mechanical ventilation with mean airway pressure ≥ 8 cmH_2_O and/or needing fractional inspiratory oxygen ≥ 0.3, II. infants requiring mechanical ventilation with mean airway pressure < 8 cmH_2_O and/or needing fractional inspiratory oxygen ≤ 0.3 with significant apnea and bradycardia, tachycardia (> 180 beats per minute) or a respiratory rate of more than 80 breaths per minute, weight gain of less than 10 g/day over 4 days, or sepsis; or III. Infants who had a hematocrit of less than 25%.

Neonatal outcomes such as IVH (using grading system proposed by Papile et al. [17]), NEC defined as modified Bell’s stage ≥ 2a [18], respiratory distress syndrome (RDS), patent ductus arteriosus (PDA), BPD defined as oxygen requirement at 28 days of age, ROP (based on ICROP staging [19]), and length of hospital stay (LOS) were determined. Morbidities including neonatal polycythemia, hyperbilirubinemia, neonatal death, maternal postpartum hemorrhage, and death were collected.

### Sample sizes

Aladangady N, et al. showed that preterm infants who received DCC for 30–45 s had increased blood volume 2–16 ml/kg and 10–28 ml/kg at cesarean and vaginal birth, respectively [16]. Hosono S, et al. demonstrated that by milking 30 cm of umbilical cord, infants could receive approximately 18 mL/kg of whole blood [20]. Hence, DCC combined with UCM may increase blood volume 2–2.5 times of DCC.

Katheria AC, et al. had 52% of the preterm infants in their DCC group who received blood transfusions [10]. We, therefore, estimated 20% of infants in the DCM-A and DCM-B groups would require blood transfusions, as calculated from DCC being combined with UCM leading to an increase in blood volume of 2–2.5 times of DCC alone. Using 80% power and a confidence value of 0.05 with two-way calculation, 40 preterm infants were required for each group.

### Statistical analysis

Demographic data was reported using percentage (%), mean, and SD median. ANOVA test was used to compare continuous variables that were normally distributed; Kruskall-Wallis was used for continuous variables not normally distributed. Pearson chi-square and Fisher’s exact test compared categorical outcome variables. *P* < 0.05 is considered significant. Data analyses were performed using an intent-to-treat basis.

## Results

**Fig. 1:**
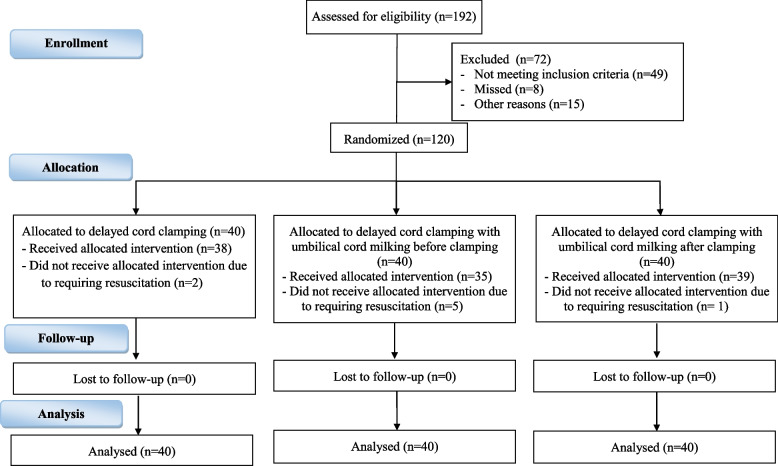
Study flow

Of the 192 preterm infants born during the study, 72 were excluded due to multiple pregnancy (*n* = 24), diagnosis with severe disabilities (*n* = 3), chromosomal abnormalities (*n* = 1), hydrops fetalis (*n* = 1), intrauterine growth retardation (*n* = 8), mother with placenta previa with hemorrhage or abruption placenta (*n* = 3), prolapsed cord (*n* = 2), fetal distress before birth (*n* = 7), birth before or on arrival (*n* = 15), and absence of consent (*n* = 8) (Fig. 1). Finally, 120 were enrolled, with 40 infants randomized into each group.

Baseline and delivery room characteristics were not different between groups, with the notable exception of GA and Apgar scores at 1 and 5 min (Table 1). Although the average gestational age was different, when looking at the proportion of preterm infants born before 32 weeks, there was no difference. Eight infants (two in the DCC group, five in DCM-B, and one in DCM-A) did not receive placental transfusion; they required neonatal resuscitation and were analyzed in their assigned groups.

**Table 1:** Baseline and delivery room characteristics

**Characteristic**	**DCC** (*n* = 40)	**DCM-B** (*n* = 40)	**DCM-A** (*n* = 40)	**p**
**Baseline characteristics**				
Maternal age, y (mean ± SD)	29.5 ± 6.0	28.3 ± 6.2	29.2 ± 7.7	0.24
Complete prenatal steroid (n, %)	21 (52.5)	14(35)	19(47.5)	0.95
Cesarean section (n, %)	24(60)	17(42.5)	17(42.5)	1.0
Male (n, %)	21(52.5)	20(50)	21(52.5)	1.00
Gestational age, wk (mean ± SD)	31.5 ± 1.7	32.1 ± 1.7	32.4 ± 1.1	0.01
GA ≤ 32 week (n, %)	29 (72.5)	23 (57.5)	24(60)	0.79
Birth weight, g (mean ± SD)	1724.3 ± 413.2	1785.3 ± 397.2	1880.6 ± 355.9	0.64
Apgar at 1 min (median)	9 (1–9)	9 (4–9)	9 (5–9)	< 0.01
Apgar at 5 min (median)	10 (3–10)	10 (5–10)	10 (7–10)	< 0.01
Apgar < 7 at 1 min (n, %)	7(17.5)	5(12.5)	2(5)	< 0.01
Apgar < 7 at 5 min (n, %)	2 (5)	1 (2.5)	0(0)	0.04
**Delivery room characteristics**				
Delivery room temperature (°C) (mean ± SD)	25.1 ± 0.8	24.9 ± 0.9	25 ± 0.6	0.1
Temperature on admission (°C) (mean ± SD)	36.9 ± 0.6	36.7 ± 0.7	36.7 ± 0.6	0.44
Temperature < 36.5 °C (n, %)	8 (20)	13 (32.5)	9 (22.5)	0.77
Required positive pressure ventilation (n, %)	8 (20)	7 (17.5)	4 (10)	0.18
Intubation in delivery room (n, %)	6 (15)	4 (10)	2 (5)	0.52

The primary outcome of the number of infants needing RBC transfusions during the first 28 days of life was not statistically significant in the DCC, DCM-B, and DCM-A groups (*p* = 0.24) as shown in Table 2. For the secondary outcomes, there were no significant differences in the prevalence of IVH (*p* = 0.22) and NEC (*p* = 0.78) as shown in Table 3. There was significantly higher BPD incidence, days for ventilation and oxygen use, as well as LOS in the DCC group as shown in Table 3. There was no infant who was diagnosed with any stage of ROP, severe IVH or died. No mothers experienced postpartum hemorrhage, or death.

**Table 2:** Hematologic outcomes

Outcomes	DCC	DCM-B	DCM-A	p
Hematocrit on admission, % (mean ± SD)	54.0 ± 5.5	53.3 ± 6.0	54.3 ± 5.8	0.88
Hematocrit at 1 week of age, % (mean ± SD)	46.0 ± 6.2	46.1 ± 6.9	46.6 ± 7.4	0.53
Hematocrit at 4 weeks of age^a^, % (mean ± SD)	33.8 ± 4.9	33.2 ± 5.9	33.5 ± 5.4	0.56
Blood loss within 28 days (ml) (mean ± SD)	19.5 ± 9.2	18.7 ± 10.2	14.9 ± 8.5	0.52
Need for red blood cell transfusion within 28 days (n, %)	10 (25)	8 (20)	5 (12.5)	0.24

^a^Results for 36 infants in the DCC group, 36 infants in the DCM-B group and 37 infants in the DCM-A group

**Table 3:** Neonatal outcomes^a^

Outcomes	DCC	DCM-B	DCM-A	p
Peak bilirubin level, mg/dL (mean ± SD)	9.5 ± 2.7	9.5 ± 2.4	9.7 ± 1.9	0.07
Phototherapy (n, %)	39 (97.5)	37 (92.5)	35 (87.5)	< 0.01
Duration of phototherapy, days (mean ± SD)	6.2 ± 3.3	5.5 ± 4.5	4.8 ± 3.2	0.16
Respiratory distress syndrome (n, %)	19 (47.5)	12 (30)	10 (25)	0.67
Surfactant (n, %)	12 (30)	8 (20)	5 (12.5)	0.13
Patent ductus arteriosus (n, %)	12 (30)	8 (20)	7 (17.5)	0.48
Bronchopulmonary dysplasia (n, %)	18 (45)	11 (27.5)	6 (15)	0.01
Ventilator, days (mean ± SD)	3.6 ± 9.2	1.6 ± 3.7	0.5 ± 1.7	< 0.01
Oxygen therapy, days (mean ± SD)	28.6 ± 30	16.1 ± 18.0	10.1 ± 14.6	< 0.01
Intraventricular hemorrhage Grade I (n, %)	12 (30)	6 (15)	13 (32.5)	0.22
Necrotizing enterocolitis (n, %)	3 (7.5)	0	4 (10)	0.78
Length of hospital stay, days (mean ± SD)	42.7 ± 29.2	31.7 ± 18.9	26 ± 15.3	0.01

^a^NO infant was diagnosed with any stage of ROP, severe IVH or died

## Discussion

UCM combined with DCC; DCM-B or DCM-A should, theoretically, increase blood volume in preterm infants, compared to DCC alone. In our study, all three techniques showed no variations in hematocrit after intervention. Preterm infants in DCM-B (20%) and DCM-A (12.5%) groups requiring blood transfusion were fewer than those in DCC (25%) group but it was not significantly different. Our study had a lower rate of infants requiring transfusion compared to Jasani B et.al, a systematic review and network meta-analysis, which showed the rate of infants needing RBC transfusion in DCC group (38.3%) compared to immediate cord clamping (ICC) group (46.9%), and UCM (32.3%) compared to ICC group (46.9%) [21]. There were two possible reasons: first, there was a difference in the GA of the infants enrolled in the study. Jasani B et.al enrolled more infants with a lower GA (GA 22^0/7^- 36 ^6/7^ weeks) than those in our study (GA 28^0/7^- 33 ^6/7^ weeks) [21]. Second, all our techniques, DCC (45 s), DCM-B and DCM-A, were different from Jasani B et.al, ICC, DCC (30–120 s), UCM followed by DCC [21]. Our practices may result in the infant receiving a greater amount of blood.

Placental transfusions also reduced the incidence and severity of IVH as well as NEC. Our study showed NEC incidence was 0—10%, averaging 5.8%, versus our previous report at 15% [22] and no infants had severe IVH. Many studies have reported the same benefits for preterm infants whether they received DCC or UCM. Shirk SK, et al. saw no statistically significant differences in occurrences of transfusion, NEC, or IVH between DCC and UCM in preterm infants who were GA 23—34 weeks 6 days [23]. Without any apparent variation in outcomes, all techniques of placenta transfusions had the same benefit. Cord blood is known to be a rich source of stem cells. This may be the reason why DCC combined with UCM has a lower incidence of BPD, and less duration of ventilation and oxygen use, as also found in previous meta-analyses [24].

No observable negative effects occurred; most importantly, there were no cases of polycythemia, or neonatal death, and no maternal postpartum hemorrhage or death. Another benefit was that hypothermia incidence was reduced by half, compared to our prior intervention period. Shirk SK, et al. reported that performing DCC or UCM in low birthweight infants made the infants warmer than immediate cord clamping as placental transfusion gives warm blood [23]. As is generally known, hyperbilirubinemia is a concern for all preterm infants. Many studies showed no differences in terms of bilirubin levels or duration of phototherapy [10, 23, 24], the same as our study.

Our study was novel in comparing DCM-B and DCM-A to DCC in a randomized controlled trial. However, our outcomes lacked statistical significance because the sample size was small. Compared to previous studies, the number of preterm infants requiring transfusions in our study was also lower. This may be due to the difference in GA; the preterm infants in our study delivered at GA of GA 28^0/7^- 33 ^6/7^ weeks, requiring fewer transfusions than the preterm infants (GA 23^0/7^- 31^6/7^ weeks) in Katheria and colleagues’ study, who needed more transfusions overall because of their extreme prematurity. Other factors that may affect the volume of placental transfusion, including uterine contractions and the onset of infants’ ventilation, were not collected and may have contributed to our results. Our predetermined effect of DCC combined with UCM was unlikely to be detected in our small sample size. Hence, a larger population is required in future studies. Nonetheless, long-term follow up for neurodevelopmental outcomes should be future research.

## Conclusion

All placental transfusion techniques, DCC and DCC combined with UCM, provided the same benefits for preterm infants born at GA of 28 and 33^6/7^ weeks in terms of reducing the need for RBC transfusions, severities of IVH and incidence of NEC without increasing comorbidity.

## Data Availability

The datasets generated during and/or analyzed during the current study are available from the corresponding author on reasonable request.

## Abbreviations

DCC: Delayed cord clamping
UCM: Umbilical cord milking
DCM-B: DCC with UCM performed before clamping
DCM-A: DCC with UCM performed after clamping
IVH: Intraventricular hemorrhage
NEC: Necrotizing enterocolitis
RBC: Red blood cell
RDS: Respiratory distress syndrome
PDA: Patent ductus arteriosus
BPD: Bronchopulmonary dysplasia
ROP: Retinopathy of prematurity
LOS: Length of hospital stay
